# A Multi-Posture Grasping Manipulator Actuated by Shape Memory Alloy with Different Functional Modules

**DOI:** 10.3390/mi15111328

**Published:** 2024-10-30

**Authors:** Xiaozheng Li, Chongjing Cao

**Affiliations:** Research Centre for Medical Robotics and Minimally Invasive Surgical Devices, Shenzhen Institute of Advanced Technology (SIAT), Chinese Academy of Sciences, Shenzhen 518055, China; xz.li@siat.ac.cn

**Keywords:** multi-posture, grasping, manipulator, module, shape memory alloy

## Abstract

Currently, multi-posture robots have complex grasping robotic manipulators with low power density, making it difficult to miniaturize and integrate. In this paper, a multi-posture grasping manipulator actuated by shape memory alloy with different functional modules is presented. It is composed of deflection, translation, rotation and grasping modules. Based on a D-H parameter method, the end motion trajectory model is established and the end motion space is drawn. Finally, the grasping experiment of a light circular object is carried out to verify the validity of the multi-posture grasping function of the multi-module combination manipulator, which provides a choice for future intelligent robot manipulators.

## 1. Introduction

Combined multi-posture manipulators offer advantages such as high flexibility, strong reconfigurability and wide adaptability [1,2,3]. They can alter the connection methods and operating states among different functional modules within the manipulators based on various tasks and requirements, thereby achieving multiple forms of transformation and motion control. Consequently, multi-posture manipulators have a wide application prospect in the fields of medical devices, aerospace and intelligent robots [4,5,6].

Traditional multi-posture grasping manipulator actuation methods, such as electric motor, hydraulic and pneumatic [7,8,9], are often larger in size and weight, which limits the portability and integration of the manipulator. With the development of intelligent drive technology, it provides a solution for miniaturized and integrated manipulators. At present, smart actuation materials such as shape memory alloy, dielectric elastomer, piezoelectric and magnetorheological fluids are widely used [10,11,12,13,14,15]. Different smart materials have their own advantages and disadvantages. Among them, shape memory alloys, as the smart materials with the best memory performance among metal materials, integrate perception and actuation, have a high power ratio, low noise, corrosion resistance, strong biocompatibility and other properties [16,17,18]. They are highly favored in the design of smart grasping manipulators. In addition, how to design a grasping manipulator that can achieve multi-posture grasping and effectively integrate shape memory alloy (SMA) into existing systems are also current research focuses.

At present, there are many multi-posture mechanical manipulators based on shape memory alloys. For example, Liu et al. [19] designed a multi-segment SMA-driven soft robotic manipulator, which achieved object grasping and obstacle avoidance. Daniela et al. [20] developed a flexible finger based on shape-memory alloy modules and introduced the basic concepts and a mathematical model for the designed device. Cortez-Vega et al. [21] presented a multi-joint snake-like manipulator that achieves a parallel antagonistic configuration of actuators for moving each joint. Choudhury et al. [22] developed a three-degree-of-freedom U-shaped fixed base planar parallel manipulator based on SMAS linear actuator, and studied the workspace and trajectory tracking capability of the proposed manipulators. Liu et al. [23] proposed a flexible robotic manipulator based on inchworm-snake for object grasping, equipped with multi-stage SMA actuators. Tuyboyov et al. [24] developed a multi-mode soft composite bending actuator based on the interweaving of glass fiber textiles interwoven with SMA wires and polydimethylsiloxane soft matrix. By changing the interlacing pattern of SMA wires, the actuator shows multi-mode bending behavior. The actuators with three different bending modes are manufactured and the prototype of the gripper is prepared, and different objects can be grasped flexibly. Kang et al. [25] proposed a multi-degree-of-freedom actuator that can simulate the trunk movement of elephants. SMA is integrated into the actuator made of soft polymer to imitate the flexible body and muscles of the elephant trunk. It realizes the bending motion of the trunk and the operation of wrapping and lifting objects. Recently, Yang et al. [26] introduced a flexible robotic manipulator with variable stiffness capability, inspired by the muscle structures of creatures/organs such as octopuses and human wrists. The manipulator is composed of three pairs of SMA springs and supercoiled polymer strings in an antagonistic configuration. By utilizing compact mixing and antagonistic actuation methods, the variable stiffness characteristics of the robotic manipulator have been further amplified. Therefore, it can be found that the existing work is basically a variety of manipulators designed with a single actuation module as the starting point, and the degree of freedom is limited. However, the manipulator actuated by SMA with multiple modules assembled and actuated separately has the characteristics of multiple degrees of freedom and multi-postures, and has the advantages of convenient carrying, installation, maintenance, lightweight and biocompatibility. So, the development of multi-posture lightweight mechanical manipulators is still needed in future portable medical surgical boxes.

Therefore, a multi-posture grasping manipulator actuated by SMA is proposed in this paper, which is composed of different functional modules based on SMA wires. Then, the structural design of the manipulator combined with each functional module is given, and its kinematics model is established. Next, the motion trajectory and space of the manipulator are analyzed. Finally, the motion range of the designed manipulator is verified by experiments. In addition, the grasping of circular lightweight objects is taken as an example to verify the effectiveness of the designed manipulator. The main contributions are as follows:

(1) Based on different functional modules actuated by SMA wires, a multi-posture manipulator is realized.
(2) The end motion trajectory space of the designed manipulator is preliminarily studied, and the light object is grasped. It lays a foundation for the design of lightweight portable manipulators in the future.

The rest of this paper is arranged as follows. Section 2 introduces the overall design and implementation of the manipulator. It includes the kinematics model of the whole structure and the analysis of the end trajectory. Section 3 is the experiments and results, which include the multi-posture motion and the grasping of circular objects. Finally, the conclusion is given in Section 4.

## 2. Design and Realization

### 2.1. Overall Structural Design and Working Principle

**Figure 1:**
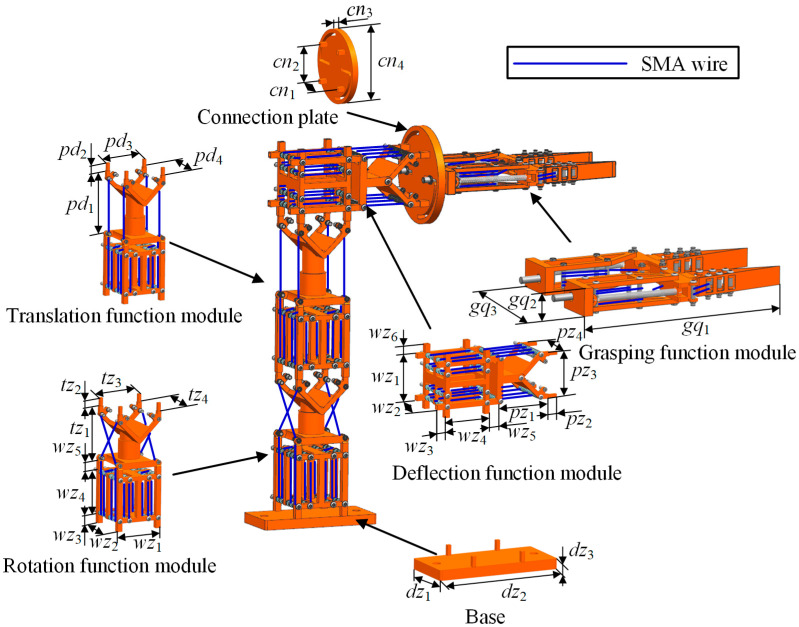
Typical multi-posture grasping manipulator schematic diagram.

The multi-posture grasping manipulator is composed of multiple SMA actuation modules with different functions. By controlling each module’s deformation state and actuation force, the grasping manipulator can achieve multi-posture motions. Based on this concept, a multi-posture grasping manipulator is designed, as shown in Figure 1; the modules were proposed in previous work [27,28,29], including winding modules, YT-type rotational and translational modules, a VT-type deflection module and a grasping module in series.

The goal is to be able to rotate left and right by 10°, move up and down by 12 mm, deflect up and down by 10°, and successfully grasp lightweight objects. According to the target requirements, the primary dimensional parameters of the multi-posture grasping manipulator are presented in Table 1.

**Figure 2:**
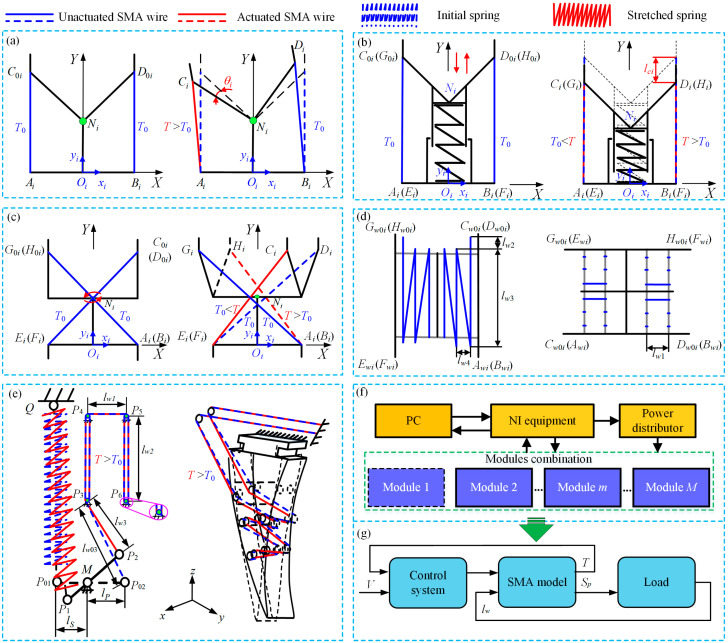
Functional implementation principles and control diagrams in different modules [27,28,29]: (**a**) deflection function module; (**b**) translation function module and (**c**) rotation function module; (**d**) winding module; (**e**) grasping function module; (**f**) schematic diagram of overall structural control and (**g**) schematic diagram of SMA actuation control for each module.

As shown in Figure 2, it is a schematic diagram of the principle of realizing deflection, translation, rotation, increasing SMA wire length and grasping when different modules are driven by SMA. Figure 2a shows that the SMA wire is arranged in an antagonistic manner on both sides. When the SMA wire is heated and actuated, it can be deflected left and right. Similarly, the translational module in Figure 2b can move up and down with the cooperation of SMA wire and compression spring. The SMA wire in Figure 2c is also in an antagonistic arrangement, and the symmetric SMA wire can rotate counterclockwise and clockwise after being heated and actuated, respectively. Figure 2d shows the arrangement of SMA in the winding module, which can increase the actuation stroke of the SMA wire. The SMA two-stage actuation gripper in Figure 2e can realize the large-scale grasping movement and fine-tuning under the combined actuation of the first and second-stage SMA wire. Figure 2f shows a schematic diagram of signal acquisition and transmission between the modules in the entire combination manipulator. The National Instruments (NI) device collects and sends signals from the Labview on the personal computer (PC), where the power supply supplies power to each module separately. Figure 2g shows the principle of SMA actuation structure in each module and the schematic diagram of the force and length change of SMA wire heated by a power supply. The temperature (*T*) of SMA wire in different modules is changed by the heating of the power supply (*V*). Subsequently, the actuation force (*S_p_*) is generated to change the structure, and then the length of SMA wire (*l_w_*) changes. Therefore, the change of the whole manipulator can be controlled by controlling the driving voltage in different modules.

### 2.2. Kinematic Model

In the kinematic analysis of the multi-posture grasping manipulator, each joint theoretically possesses 6 degrees of freedom relative to the preceding joint and is represented using transformation matrices. The forward kinematic equations are obtained by sequentially multiplying the transformation matrices of each joint, thereby solving for the position and orientation of its end-effector in the base coordinate system. However, considering the constrained relationships between the joints, employing such complex descriptive forms is unnecessary. Here, the Denavit–Hartenberg parameter model (D-H model) proposed by Denavit and Hartenberg [30] in 1955 for robots is adopted to analyze the structural linkage and joint models.

**Figure 3:**
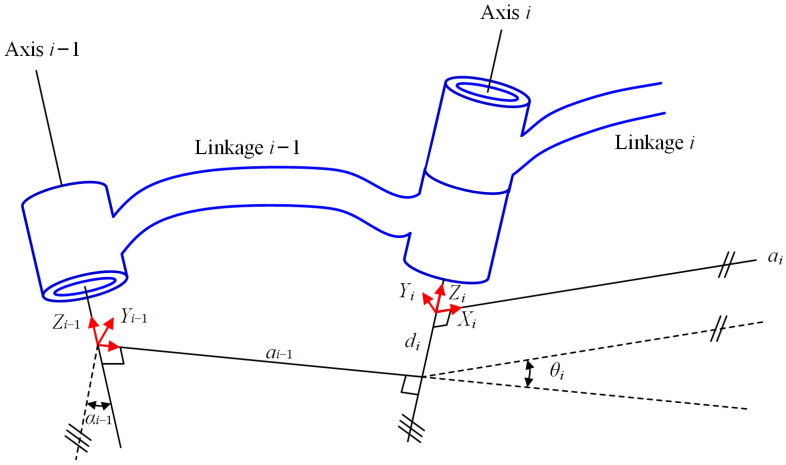
Schematic diagram of two adjacent links of the multi-posture manipulator [30,31]. Adapted with permission from Refs. [30,31].

As depicted in Figure 3, two adjacent linkages can be described as the *i* − 1th joint, the *i* − 1th linkage, the *i*th joint and the *i*th linkage. According to the coordinate system, coordinate system {*A_i_*_−1_} is fixed on the *i* − 1th linkage and coordinate system {*A_i_*} is fixed on the ith linkage. The transformation matrix from coordinate system {*A_i_*_−1_} to coordinate system {*A_i_*} can be established through the following four steps:

Step 1: Place the initial coordinate system in coordinate system {*A_i_*_−1_}, and rotate it around the *X_i_*_−1_ axis by *α_i_*_−1_ to make the *Z_i_*_−1_ axis parallel to the *Z_i_* axis.

Step 2: Translate the coordinate system along the *X_i_*_−1_ axis by *a_i_*_−1_ to align the *Z_i_*_−1_ collinear with the *Z_i_* axis.

Step 3: Rotate the coordinate system around the *Z_i_*_−1_ axis (*Z_i_* axis) by *θ_i_* to make the *X_i_*_−1_ axis parallel to the *X_i_* axis.

Step 4: Translate the coordinate system along the *Z_i_*_−1_ axis (*Z_i_* axis) by *d_i_* to transform it to {*A_i_*}.

The next step of transformation is carried out on the coordinate system after these four transformations, that is, right multiply the matrix to obtain the transformation matrix from coordinate system {*A_i_*_−1_} to coordinate system {*A_i_*}:

(1) Tii−1=RXαi−1Trai−1,0,0RZZ,θiTr0,0,di

where,

$$R_{X}(\alpha )=\left[\begin{array}{cccc} 1 & 0 & 0 & 0\\ 0 & \cos\alpha & -\sin\alpha & 0\\ 0 & \sin\alpha & \cos\alpha & 0\\ 0 & 0 & 0 & 1 \end{array}\right],$$

$$R_{Z}(\theta )=\left[\begin{array}{cccc} \cos\theta & -\sin\theta & 0 & 0\\ \sin\theta & \cos\theta & 0 & 0\\ 0 & 0 & 1 & 0\\ 0 & 0 & 0 & 1 \end{array}\right],$$

$$T_{r}(a,c,d)=\left[\begin{array}{llll} 1 & 0 & 0 & a\\ 0 & 1 & 0 & c\\ 0 & 0 & 1 & d\\ 0 & 0 & 0 & 1 \end{array}\right].$$

This further leads to:

Tii−1=RXαi−1Trai−1,0,0RZθiTr0,0,di=cosθi−sinθi0ai−1sinθicosαi−1cosθicosαi−1−sinαi−1−sinαi−1disinθisinαi−1cosθisinαi−1cosαi−1cosαi−1di0001

Eventually, the homogeneous transformation matrix from the base coordinate system {*A*_0_} to the end-effector coordinate system {*A_n_*} can be derived:

(2) Tn0=T10T21⋯Tnn−1

Upon obtaining Tn0=Rn0000Pn01, the upper-left 3 × 3 matrix Rn0 represents the rotation matrix of the end-effector in the base coordinate system, while the vector Tn0=Rn0000Pn01 denotes the spatial position coordinates of the end-effector in the base coordinate system.

Given the control parameters (angles or displacements) of each joint, the motion trajectory of the multi-posture grasping manipulator, composed of rotational, translational and deflection modules in series can be solved through forward kinematic analysis. Although the multi-posture grasping manipulator employs a limited number of functional modules, its geometric parameters remain relatively complex. The Denavit–Hartenberg (D-H) model establishes a coordinate system for each linkage based on predefined rules, facilitating the description of the transformation relationship between adjacent coordinate systems. Essentially, it decomposes the transformation between adjacent coordinate systems into several steps, each with a single parameter. Therefore, the first step is to abstract each module as an equivalent linkage-joint form and then fix a coordinate system on each linkage. By describing the relationships between these linkage coordinate systems, the end-effector pose can be linked to the joint variables.

**Figure 4:**
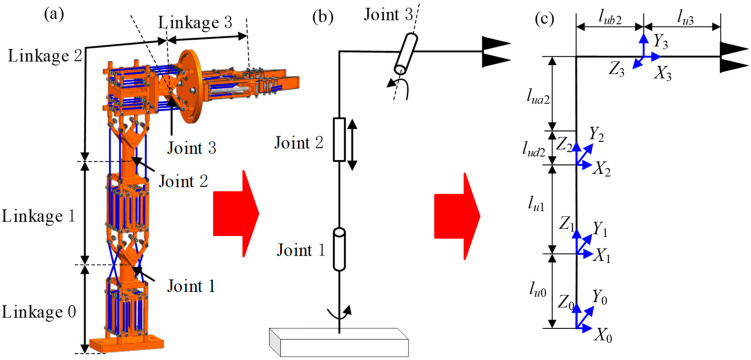
Simplified kinematic model of a multi-posture grasping manipulator: (**a**) geometric model; (**b**) abstract linkage joint model; and (**c**) kinematics model.

Before conducting kinematic analysis on the multi-posture grasping manipulator, the actual existing functional modules are abstracted into kinematic models. From the geometric model in Figure 4a, they are abstracted into the linkage-joint model in Figure 4b. Subsequently, the kinematic model shown in Figure 4c can be established. When determining the axes, the *Z*-axis represents the rotation axis of the linkage joint, while the *X*-axis is the perpendicular bisector between the *Z*-axis of the current joint and the next joint (upwards). The base coordinate system is located on the base of the multi-posture manipulator (linkage 0). The parameters of the linkages used are listed in Table 2.

Once the coordinates are determined, a D-H table can be established by analyzing the relationships between equivalent linkages to determine the four parameters mentioned above, as shown in Table 3. Here, *θ_min_*_1_, *θ_max_*_1_, *θ_min_*_3_ and *θ_max_*_3_ represent the minimum and maximum angles that rotational joints 1 and 3 can rotate.

### 2.3. End Motion Trajectory Space Analysis

**Figure 5:**
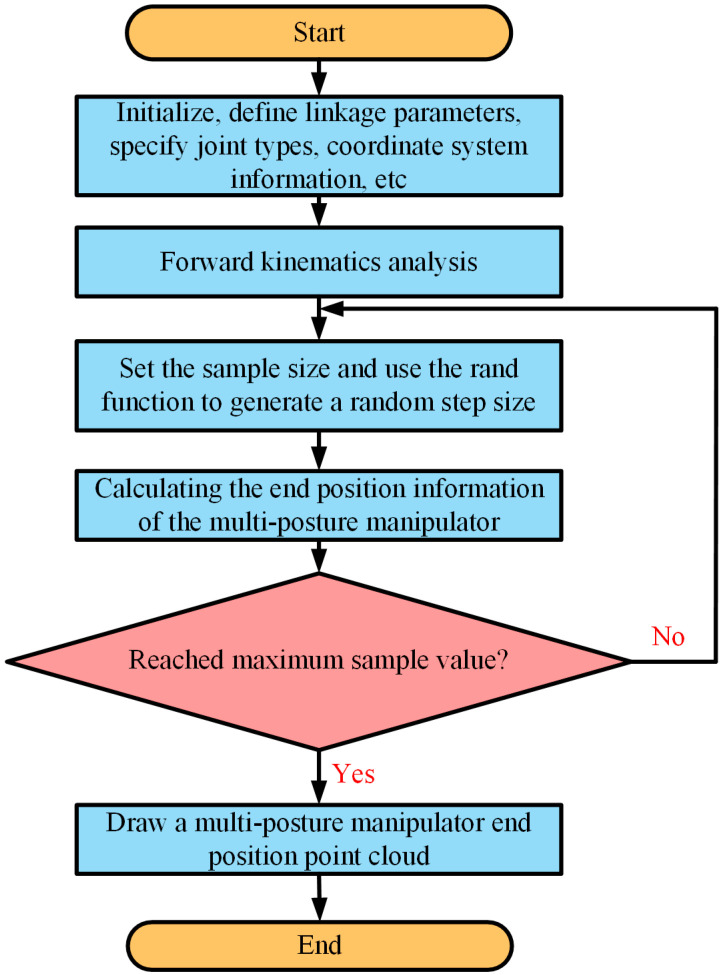
The analysis process of end motion trajectory space of the multi-posture grasping manipulator.

**Figure 6:**
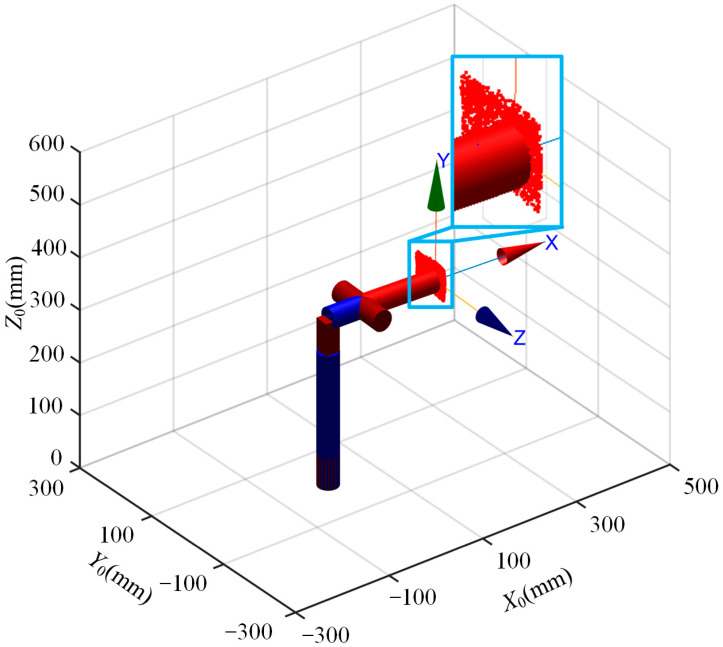
Multi-posture grasping manipulator end motion trajectory space.

The end motion trajectory space of the multi-posture grasping manipulator consists of the set of all reachable positions of the end-effector, and its shape and size play a crucial role in guiding the rational design of the multi-posture grasping manipulator’s structure. Compared with grid-based methods and geometric methods, the Monte Carlo method is often employed for solving the workspace of the multi-posture grasping manipulator due to its strong graphical display capabilities, fast computation speed and simplicity of operation [32]. By solving the forward kinematics, corresponding end-effector positions can be obtained, and visualizing a lot of end-effector position points enables visualization of the end motion trajectory space. The solution steps are as follows in Figure 5:

Motion space analysis refers to the analysis and calculation of relevant parameters of the multi-posture grasping manipulator during its motion process, in order to understand its motion characteristics and provide references for optimization design and control. Below is the procedure to draw the motion space of the multi-posture grasping manipulator composed of rotational, translational, deflection and gripping SMA drive modules. Where *θ_min_*_1_, *θ_max_*_1_, *θ_min_*_3_ and *θ_max_*_3_ are set to −10°, 10°, −10° and 10°, respectively, and the translational distance *l_ud_*_2_ is set to 12 mm based on the previous chapters’ work. According to task requirements, the end-effector motion trajectory space of the multi-posture grasping manipulator is depicted as shown in Figure 6.

From the simulation results in Figure 6, it can be concluded that the multi-posture grasping manipulator can achieve the predetermined objectives of upward and downward deflection of 10°, upward and downward translational motion of 12 mm and left and right rotation of 10°.

## 3. Experiments and Results

### 3.1. Preparation of Experimental Specimens

**Figure 7:**
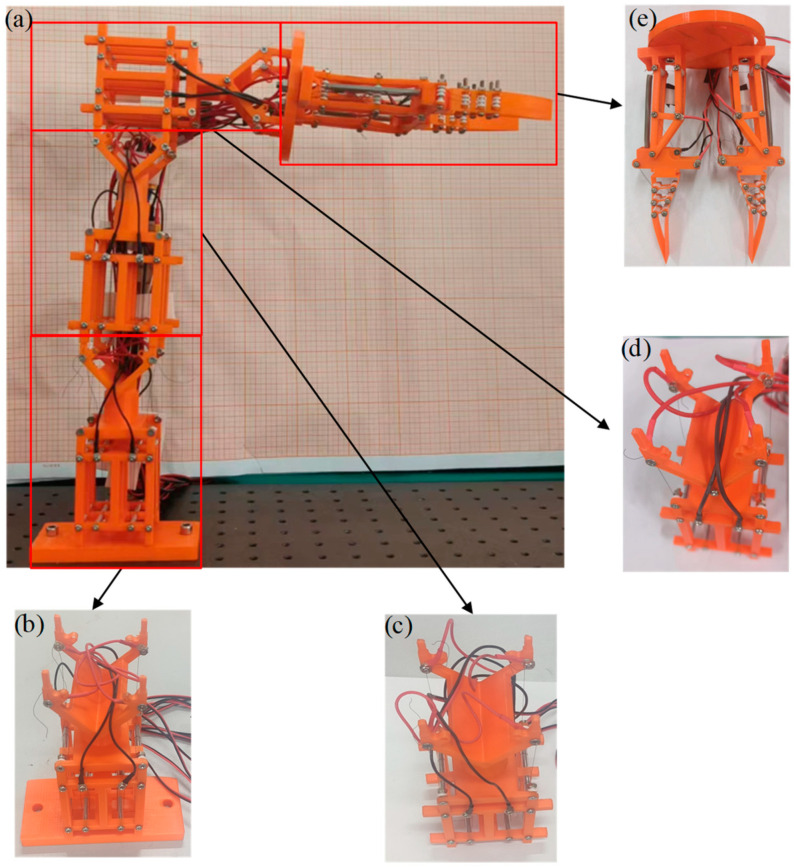
Test pieces for multi-posture grasping manipulator and each functional module: (**a**) the multi-posture grasping manipulator; (**b**) the base with rotational function module; (**c**) the translational function module; (**d**) the deflection function module; and (**e**) the connection plate with grasping function module.

The components such as deflection actuation module, translational/rotational actuation module, grasping actuation module, winding module, base and connecting plate are fabricated by a 3D printer, as shown in Figure 7. The radius of the SMA wire is 0.1 mm, and denoted as *r_wire_*. Based on the fabricated modules, in which three strands of SMA wire are installed in the coiling module and one strand in other modules, the multi-posture grasping manipulator depicted in Figure 7 is assembled.

### 3.2. Experimental Verification and Analysis of the End Motion Space

**Figure 8:**
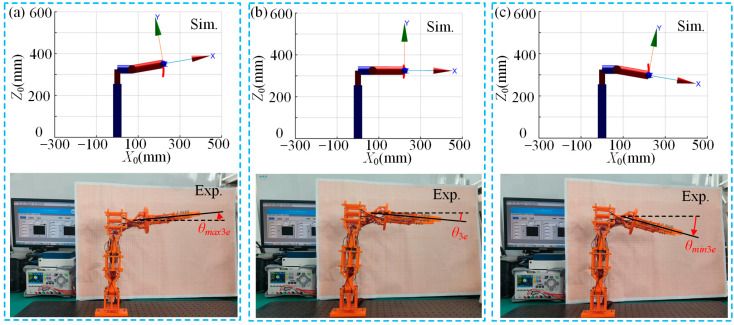
Simulation and experimental comparison of multi-posture grasping manipulator deflection function module during actuation: (**a**) upward deflection; (**b**) initial state; and (**c**) downward deflection.

Figure 8 shows a comparison between the simulation and experimental results of the multi-posture grasping manipulator’s deflection module during upward deflection, initial state and downward deflection. The angles for upward deflection (*θ_max_*_3*e*_), initial state (*θ*_3*e*_) and downward deflection (*θ_min_*_3*e*_) are measured at 6.3°, 5.2° and 16.0°, respectively. From Figure 8a–c, it can be observed that the experimental and simulated motion trends are generally consistent when the deflection module is actuated. The basic functionality of achieving the predetermined 10° upward and downward deflections is realized. However, due to factors such as gravity and friction, the experimental results show that in the initial state, the end-effector of the multi-posture grasping manipulator exhibits a downward deflection. Additionally, during deflection module actuation, the upward deflection angle is slightly smaller, while the downward deflection angle is larger. This asymmetry is attributed to the effect of gravity: during upward deflection, the manipulator needs to overcome gravity, while during downward deflection, the effect of gravity aids in increasing the deflection angle. Consequently, this phenomenon results in an asymmetry in the upward and downward deflection angles.

**Figure 9:**
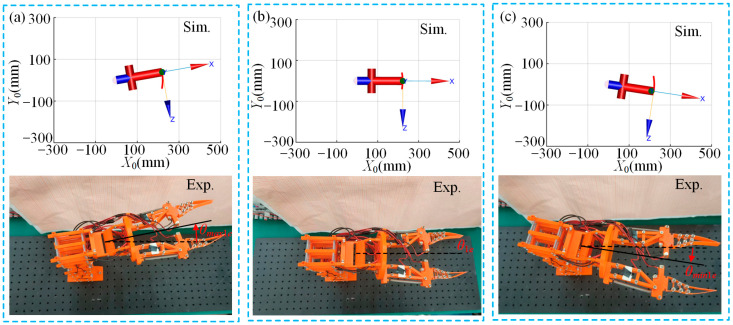
Simulation and experimental comparison of the multi-posture grasping manipulator rotating (translational and rotational) function module during actuation: (**a**) counterclockwise rotation; (**b**) initial state; and (**c**) clockwise rotation.

Figure 9 illustrates a comparison between simulation and experimental results of the multi-posture grasping manipulator’s rotation (translational and rotational) functionality module. The angles for counterclockwise rotation (*θ_max_*_1*e*_), initial state (*θ*_1*e*_) and clockwise rotation (*θ_min_*_1*e*_) are measured at 9.6°, 0° and 9.9°, respectively. From Figure 9a–c, it is evident that the experimental results are consistent with the simulation results when the rotation (translational and rotational) functionality module is actuated. The basic functionality of achieving the predetermined counterclockwise and clockwise rotations of 10° is realized, with errors within a reasonable range. This indicates that the rotation functionality module is almost unaffected by gravity during actuation. This is because the rotation functionality module rotates about the *Z*_1_ axis, which is aligned with the direction of gravity. Additionally, the effect of gravity only increases the friction in the rotation joint, and the increase in friction in the rotation joint is relatively minimal and can be neglected. Therefore, gravity has almost no effect on the rotational angle in the rotation direction.

**Figure 10:**
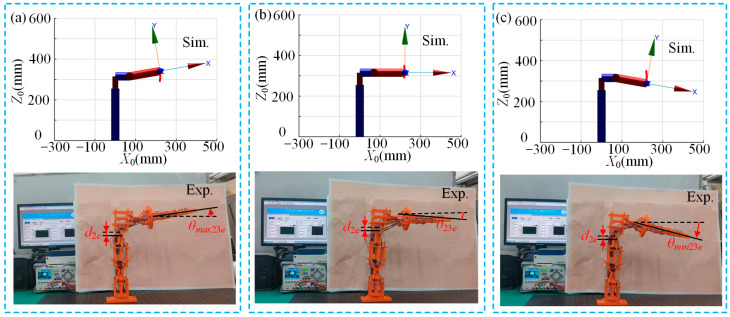
Simulation and experimental comparison of the multi-posture grasping manipulator deflection function module during actuation: (**a**) translation down + upward deflection; (**b**) initial state; and (**c**) translation down + downward deflection.

Figure 10 presents a comparison between the simulation and experimental results of the multi-posture grasping manipulator’s translational and deflection functionality modules. The translational displacement (*d*_2*e*_), upward deflection angle (*θ_max_*_23*e*)_, initial state angle (*θ*_23*e*_) and downward deflection angle (*θ_min_*_23*e*_) are measured at 10.6 mm, 7.2°, 6.0° and 15.3°, respectively. From Figure 10a–c, it is observed that the basic functionality of achieving the predetermined 12 mm upward and downward translational motion and 10° deflection is realized when the translational and deflection functionality modules are actuated. However, the translational displacement in the experiment is slightly smaller compared to the simulation results, which can be attributed to factors such as gravity, friction and human error. When the translational and deflection functionality modules are actuated together, the deflection angle is consistent with that when only the deflection functionality module is actuated, indicating that the influence of the translational functionality module on the deflection angle output of the deflection functionality module is minimal.

**Figure 11:**
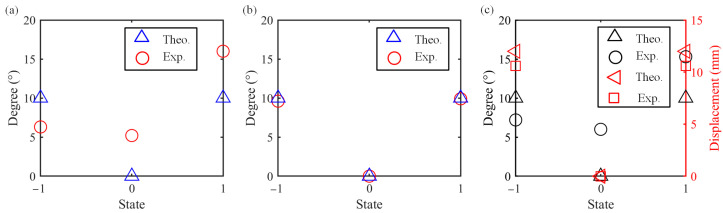
Simulation and experimental comparison of multi-posture grasping manipulator: (**a**) deflection; (**b**) rotation; and (**c**) translation down with deflection.

The downward deflection, counterclockwise rotation and downward translation with upward deflection in the three postures are represented by “−1”. The initial state is represented by “0”. The upward deflection, clockwise rotation and downward translation with downward deflection are represented by “1”. The theoretical reference values and experimental results are plotted in Figure 11. From Figure 11, it can be found that the experimental results are consistent with those in Figure 8, Figure 9 and Figure 10, but there is still a certain gap with the theoretical values, especially in the process of up-and-down deflection and translation down movement. This is partly caused by the gravity of the module itself, and partly by friction and errors in manual installation. In counterclockwise and clockwise rotation, the error is relatively small due to the small influence of factors such as gravity.

**Figure 12:**
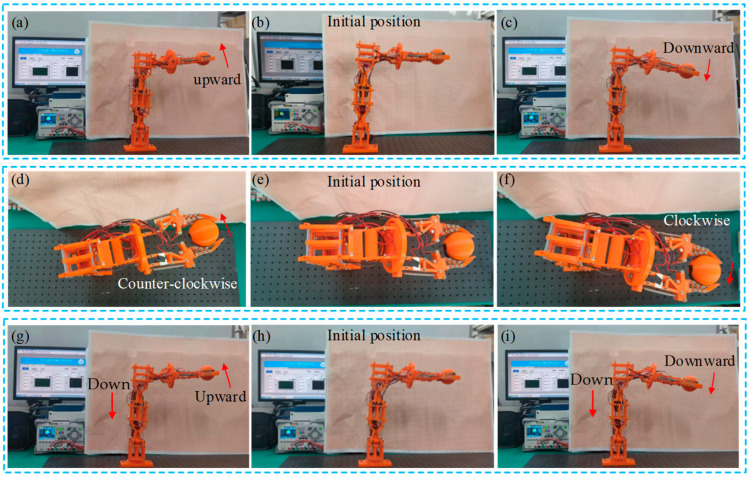
The multi-posture grasping manipulator grasping a ball under the actuation of different functional modules: (**a**) upward deflection; (**b**) initial state; (**c**) downward deflection; (**d**) counterclockwise rotation; (**e**) initial state; (**f**) clockwise rotation; (**g**) translation down + upward deflection; (**h**) initial state; and (**i**) translation down + downward deflection.

Figure 12 depicts the experimental results of grasping a lightweight sphere with a radius of 58 mm and a mass of 20 g actuated by different functional modules. Under the same conditions, it is observed that the spatial reach achieved by the different actuation modules remains essentially unchanged when grasping the lightweight sphere. Additionally, the output displacement and angle of the multi-posture grasping manipulator during grasping the object also remain essentially unchanged. This indicates that under the specified parameters, the designed multi-posture grasping manipulator has a certain grasping capability and meets its basic requirements.

In conclusion, the basic functions of the manipulator with multiple modular combinations actuated by SMA wires have been basically realized; however, due to the limited power of the driving power and the length of SMA wires used, the basic stroke of deflection, translation and rotation is small, and the performance of manipulator can be enhanced by replacing the better power supply and longer SMA wires. In order to clarify the advantages and disadvantages of this design manipulator and other work, the comparative analysis is shown in Table 4. From Table 4, it can be found that the manipulator has advantages in combination and flexibility. Although pneumatic has good expansibility and modularity, its additional equipment will affect its portability.

## 4. Conclusions

This paper addresses the diverse functional requirements, including deflection, translational/rotational motion and grasping, for multi-posture grasping manipulators. A multi-posture grasping manipulator based on the combination of multi-functional actuation modules is proposed. The forward kinematic model is established using simplified links and joints, and solved using the D-H parameter method. The workspace of the manipulator’s end is visualized using the Monte Carlo method. The accuracy of the manipulator under different functional modules is verified through simulation and experimentation. Furthermore, the performance of the multi-posture grasping manipulator in grasping spherical objects under different functional module operations is analyzed through experiments, validating its various capabilities. The main results show that:

1. The forward kinematic model of the multi-posture grasping manipulator is established based on the D-H parameter method, establishing the relationship between the end-effector position vector and the joints. The accuracy of the model is verified through comparative analysis between simulation and experimentation.
2. The reachable workspace point cloud of the end-effector motion trajectory of the multi-posture grasping manipulator is obtained using the Monte Carlo method, determining the motion range of the manipulator. It basically achieves the function of rotating 10° left and right, moving 12 mm up and down, deflecting 10° up and down and successfully grasping lightweight objects.

This work lays a foundation for the development of portable lightweight medical surgical boxes in the future.

## Figures and Tables

**Table 1:** The main dimension parameters of the multi-posture grasping manipulator.

Symbol	Description	Value (mm)	Symbol	Description	Value (mm)
*dz*_1_	The width of the base	50	*pd*_2_	The height of the connecting end of the translation structure	10
*dz*_2_	The length of the base	100	*pd*_3_	The length of the translation structure	60
*dz*_3_	The height of the base	10	*pd*_4_	The width of the translation structure	60
*wz*_1_	The length of the winding module	60	*pz*_1_	The height of the deflection structure	60
*wz*_2_	The width of the winding module	60	*pz*_2_	The height of the connection end of the deflection structure	10
*wz*_3_	The height of the lower connection end of the winding module	10	*pz*_3_	The length of the deflection structure	60
*wz*_4_	The height of the winding module	60	*pz*_4_	The width of the deflection structure	60
*wz*_5_	The height of the connection end on the winding module	10	*cn*_1_	The horizontal distance of the connection point of the connecting plate	50
*wz*_6_	The height of the connection end on the winding module side	10	*cn*_2_	The vertical distance of the connection point of the connecting plate	50
*tz*_1_	The height of the rotating structure	70	*cn*_3_	The thickness of the connecting plate	8
*tz*_2_	The height of the connection end of the rotating structure	10	*cn*_4_	The diameter of the connecting plate	100
*tz*_3_	The length of the rotating structure	60	*gq*_1_	The length of the grasping structure	185
*tz*_4_	The width of the rotating structure	60	*gq*_2_	The height of the grasping structure	20
*pd*_1_	The height of the translation structure	82	*gq*_3_	The width of the grasping structure	110

**Table 2:** Equivalent link parameters of each functional module.

Symbol	Description	Value (mm)	Symbol	Description	Value (mm)
*l_u_*_0_	Length of equivalent linkage 0	95	*l_ua_*_2_	The length of the equivalent part connecting linkage 2	80
*l_u_*_1_	The length of the equivalent connecting linkage 1	140	*l_ub_*_2_	Equivalent to the length of the other part of the connecting linkage 2	70
*l_ud_*_2_	The distance of the translation	12	*l_u_*_3_	The length of the equivalent connecting linkage 3	153

**Table 3:** D-H parameters.

*i*	*α*_*i*−1_	*α*_*i*−1_	*d_i_*	*θ_i_*
1	0	0	*l_u_*_1_ *+ l_u_*_1_	*θ_min_*_1_ *~θ_max_*_1_
2	90°	*l_ub_*_2_	*l_ua_*_2_*~l_ua_*_2_ *+ l_ud_*_2_	0
3	0	*l_u_*_3_	0	*θ_min_*_3_ *~θ_max_*_3_

**Table 4:** Comparison of the proposed manipulator and the existing ones.

Reference	Actuation Source	Portability	Scalability	Modularity
[33]	Hydraulic	Poor	Poor	No
[34]	Wire-driven	Poor	Poor	No
[35]	Motor	Poor	Poor	No
[36]	Pneumatic	Medium	Good	Yes
This work	SMA	Good	Good	Yes

## Data Availability

The data that support the findings of this study are available from the corresponding authors upon reasonable request.
